# TIM-4 in macrophages contributes to nasal polyp formation through the TGF-β1–mediated epithelial to mesenchymal transition in nasal epithelial cells

**DOI:** 10.3389/fimmu.2022.941608

**Published:** 2022-08-05

**Authors:** Danxue Qin, Peiqiang Liu, Huiqin Zhou, Jing Jin, Wanyang Gong, Kunyu Liu, Siyuan Chen, Jingyu Huang, Wenjun Fan, Zezhang Tao, Yu Xu

**Affiliations:** ^1^ Department of Otolaryngology-Head and Neck Surgery, Renmin Hospital of Wuhan University, Wuhan, China; ^2^ Research Institute of Otolaryngology-Head and Neck Surgery, Renmin Hospital of Wuhan University, Wuhan, China

**Keywords:** TIM-4, macrophage, TGF-β1, EMT, CRSwNP

## Abstract

Chronic rhinosinusitis with nasal polyps (CRSwNP) is caused by prolonged inflammation of the paranasal sinus mucosa. The epithelial to mesenchymal transition (EMT) is involved in the occurrence and development of CRSwNP. The T-cell immunoglobulin domain and the mucin domain 4 (TIM-4) is closely related to chronic inflammation, but its mechanism in CRSwNP is poorly understood. In our study, we found that TIM-4 was increased in the sinonasal mucosa of CRSwNP patients and, especially, in macrophages. TIM-4 was positively correlated with α-SMA but negatively correlated with E-cadherin in CRS. Moreover, we confirmed that TIM-4 was positively correlated with the clinical parameters of the Lund-Mackay and Lund-Kennedy scores. In the NP mouse model, administration of TIM-4 neutralizing antibody significantly reduced the polypoid lesions and inhibited the EMT process. TIM-4 activation by stimulating with tissue extracts of CRSwNP led to a significant increase of TGF-β1 expression in macrophages *in vitro*. Furthermore, coculture of macrophages and human nasal epithelial cells (hNECs) results suggested that the overexpression of TIM-4 in macrophages made a contribution to the EMT process in hNECs. Mechanistically, TIM-4 upregulated TGF-β1 expression in macrophages *via* the ROS/p38 MAPK/Egr-1 pathway. In conclusion, TIM-4 contributes to the EMT process and aggravates the development of CRSwNP by facilitating the production of TGF-β1 in macrophages. Inhibition of TIM-4 expression suppresses nasal polyp formation, which might provide a new therapeutic approach for CRSwNP.

## Introduction

Chronic rhinosinusitis (CRS) is considered a complex disorder that is caused by multiple factors and mechanisms, it is a locally aggravated immune response in the nasal mucosa. According to the European position paper on rhinosinusitis and nasal polyps 2020 (EPOS 2020), CRS is classified into the following two phenotypes: chronic rhinosinusitis with nasal polyps (CRSwNP) and chronic rhinosinusitis without nasal polyps (CRSsNP) (1). According to the degree of eosinophil infiltration, CRSwNP can be further divided into eosinophilic CRSwNP (E-CRSwNP) and non-eosinophilic CRSwNP (NE-CRSwNP). Previous studies have suggested that the pathological features of CRS are associated with epithelial barrier dysfunction, host immune response, and tissue remodeling. These features mainly manifest as damaged epithelial cells in the nasal cavity and sinuses, goblet cells hyperplasia, increased extracellular matrix and fibrosis in the lamina propria, and extensive infiltration of inflammatory cells (2). However, the related mechanism is still not completely clear.

EMT often occurs during airway remodeling. It has been reported that EMT is involved in the occurrence and development of CRSwNP (3–6). During EMT, epithelial cells lose polarity, downregulate connexins, reorganize the cytoskeleton, and acquire a mesenchymal phenotype (7, 8). The chronic inflammatory state induces the loss of the epithelial adhesion molecule E-cadherin and accelerates nasal polypogenesis (9). Several inflammatory mediators have been implicated in the regulation of EMT in CRSwNP, such as transforming growth factor-beta 1 (TGF-β1) (10), matrix metalloproteinases (MMPs) (11–13), and vascular endothelial growth factor (14). These mediators can be secreted by macrophages and play an important role in EMT. Current studies have confirmed that the number of macrophages is significantly increased in CRS and is positively correlated with the severity of nasal polyps (15, 16). However, the mechanism, underlying the relationship between macrophages and CRSwNP, needs to be further explored.

Numerous studies have reported that TIM-4 is a novel molecule that is closely related to conditions of chronic inflammation, such as gastroenteritis (17), atopic dermatitis (18), and asthma. For example, in a mouse model of asthma, stimulation with the cigarette smoke extract and the cockroach allergen BLAG7 upregulated the expression level of TIM-4 and increased the expression of Th2-type inflammatory response (19, 20). Furthermore, TIM-4 is selectively expressed in antigen-presenting cells (APCs), particularly in macrophages, but not in T or B cells (21–23). The limitation of these features indicates that TIM-4 may play an important role in the regulation of macrophage function and participate in immune responses (24, 25). To date, the expression level and the role of TIM-4 in the occurrence of CRSwNP are still unclear. Given the term “combined airway disease”, we suspect that TIM-4 may also have an association with inflammation in CRSwNP. Thus, it would be very interesting to explore whether TIM-4 in macrophages participates in the EMT of CRSwNP.

## Materials and methods

### Human subjects

All participants in the study were recruited from the Department of Otolaryngology-Head and Neck Surgery, Renmin Hospital of Wuhan University (Wuhan, China), between January 2021 and June 2021. This research was approved by the Ethics Committee of Renmin Hospital of Wuhan University (No. WDRY2021-K084). Before all experiments, informed consent was obtained from all the patients and their families. For all enrolled patients, the diagnosis of CRSwNP or CRSsNP was made according to the EPOS 2020 (1). Patients with any of the following criteria were excluded: 1) younger than 18 years old; 2) diagnosed with primary ciliary dyskinesia, posterior nostril polyps, fungal rhinosinusitis, cystic fibrosis, or a systemic coagulation disorder; 3) aspirin-sensitivity; and 4) treated with antibiotics, glucocorticoids, or immune-modulating drugs for 4 weeks before surgery. The tissues of the control group were obtained from patients with only a deviated nasal septum and no other nasal diseases. In our study, mucosal tissues from the inferior turbinates of control subjects (n=14) and the uncinate processes of subjects CRSsNP (n=12), NP of E-CRSwNP (n=13), and NE-CRSwNP (n=16) were collected during endoscopic sinus surgery (ESS). Human nasal epithelial cells (hNECs) were also obtained during ESS. E-CRSwNP was defined when the percentage of eosinophils in nasal mucosa exceeded 10% of the total infiltrating cells (26). The demographic and clinical information of all subjects were summarized in **Table 1**.

**Table 1:** Characteristics of subjects.

	Control	CRSsNP	E-CRSwNP	NE-CRSwNP
Total no. of subjects	14	12	13	16
Tissue used	IT	UP	NP	NP
Age (y), mean (SD)	29 (10)	40 (14)	39 (14)	43 (14)
Asthma, (No.)	0	0	0	0
Aspirin sensitivity (No.)	0	0	0	0
Nasal steroid, (No.)	0	0	0	0
Lund–Mackay CT score, mean (SD)	-	3.7 (2.4)	9.5 (3.8)	7.7 (3.5)
Lund–Kennedy score, mean (SD)	–	1.5 (1.0)	3.6 (1.3)	3.3 (1.4)
VAS score, mean (SD)	-	13.4 (8.1)	17 (7.6)	12.4 (11.4)
Tissue dissolution, (No.)	6 (5 males)	6 (4 males)	6 (5 males)	6 (1 male)
Morphology/PCR, (No.)	8 (7 males)	11 (6 males)	11 (8 males)	9 (7 males)
Tissue extracts, (No.)	4 (4 males)	4 (4 males)	4 (4 males)	4 (4 males)

### Murine NP model and related experiments

Wild-type C57BL/6J mice (6–8 weeks old, male or female) were provided by Beijing Weitonglihua Experimental Animal Technology Co. Ltd. and housed in a specific pathogen-free facility in the Laboratory Animal Center Renmin Hospital of Wuhan University. All experimental procedures were approved by the Animal Ethics Committee of Renmin Hospital of Wuhan University (License No. WDRM 20211005). The mice were divided into four groups: the PBS group (n=6), NP group (OVA+SEB, n=7), TIM-4 mAb group (OVA+SEB+TIM-4 mAb, n=5), and dexamethasone group (OVA+SEB+DXM, n=6). A murine NP model was generated according to a previously established protocol (3, 27). Briefly, the mice in the experimental groups were sensitized by an intraperitoneal (i.p.) injection of ovalbumin (25 μg, OVA, Sigma-Aldrich) in 200 μl of PBS solution containing aluminum hydroxide (2 mg) on day 0 and day 5. Then, they were subsequently challenged with 20 μl of 6% OVA for 13 consecutive weeks *via* intranasal (i.n.) administration. During the last 8 weeks, the experimental mice were challenged with staphylococcal enterotoxin B (10 ng SEB, Toxin Technology, USA) and OVA by i.n. administration. In the experimental groups, dexamethasone (1 mg/kg) or TIM-4 mAb (0.3 mg, BioXcell, # BE0171, USA) was administered *via* i.p. or intravenous (i.v.), respectively. The PBS group was given PBS during the process as a control. The mice were euthanized and dissected at 24 h after the final challenge. The serum of mice was collected. The nasal lavage fluid of mice was collected and centrifuged after lavage with 500 µl of cold normal saline three times. Serum and nasal lavage fluid were stored at -80°C for further studies. The heads of mice were completely resected for fixation, decalcification, and paraffin embedding.

### Cell culture

Primary hNECs were obtained from patients with deviated nasal septum during ESS and cultured in BEBM medium (Lonza, Switzerland) as previously described (28). The upper airway epithelial cell line RPMI 2650 and THP-1 cells were purchased from Beina Cell Collection (Beijing, China) and maintained in RPMI-1640 medium (Gibco, USA), supplemented with 10% FBS (Gibco, USA) and 1% penicillin–streptomycin in an incubator at 37°C and 5% CO_2_. The differentiation of THP-1 cells to macrophages was induced by treatment with 100 ng/ml O-tetradecanoylphorbol-13-acetate (PMA, Sigma-Aldrich) for 48 h, and the differentiated macrophages were treated with human nasal tissue extracts (20 μg/ml) from patients with CRSwNP and CRSsNP and healthy subjects, ROS inhibitor YCG063 (10 µM, Sigma-Aldrich) and p38 MAPK inhibitor SB203580 (10 µM, Sigma-Aldrich) for 24 h. The THP-1 cells and supernatants were collected for further analyses.

### Transfection

TIM-4 knock-down (shTIM-4) in THP-1 cells was achieved by transfecting the cells with the GV493 lentivirus vector (GeneChem, China). Overexpression of TIM-4 (OE TIM-4) was achieved *via* the GV492 lentivirus vector (GeneChem, China). Lentiviral particles were transduced into THP-1 cells at a multiplicity of infection (MOI) of 10, together with HitransG-P for 72 h. The transfection efficiency was assessed by Western blot analysis. THP-1 cells stably transfected with TIM-4 were screened using puromycin for further experiments.

### Coculture procedures

Macrophages treated with OE TIM-4 were cocultured with hNECs or RPMI 2650 cells in a transwell cell culture dish (Corning, USA). The hNECs or RPMI 2650 cells were cultured on the upper inserts, and macrophages were cultured on the bottom layer. Macrophages and hNECs or RPMI 2650 cells were cocultured and treated with a TGF-β1 inhibitor SB431542 (10 µM, Sigma-Aldrich) for 24 h. Then hNECs and RPMI 2650 cells were harvested for further analyses.

### H&E and PAS staining

Nasal mucosal tissues from patients and mice were fixed, embedded and made into paraffin sections. After rehydration, the tissue sections were stained with hematoxylin and eosin. The number of polypoid lesions in the mucosa of mice was enumerated by using microscope. The polypoid lesions were characterized by the greater epithelial thickness of nasal mucosa, an inflammatory cells infiltration, edematous stroma, and the microcavities as described in previous studies (3, 29, 30). The specific characteristics of goblet cells in mouse nasal mucosa were stained using periodic acid-Schiff (PAS). All results were observed and photographed by microscopy and further analyzed by Image-Pro Plus version 6.0 and Image J software.

### Immunohistochemical staining

The tissue sections from patients and mice were deparaffinized and rehydrated. Endogenous peroxidase was blocked by 3% H_2_O_2_ for 10 min, and the antigens were retrieved in boiling 10 mM citrate buffer for 5 min. Then, they were incubated in 10% goat serum to block nonspecific antigens at room temperature. Then, the sections were incubated with antibodies against TIM-4 (CST, USA, 1:100), E-cadherin (Proteintech, China, 1:2000), α-SMA (Proteintech, China, 1:2000), or Vimentin (Proteintech, China, 1:5000) overnight at 4°C. On day 2, the sections were incubated with a horseradish peroxidase-conjugated secondary antibody at room temperature for 30 min and then developed with DAB solution. All results were observed and photographed by microscopy and further analyzed by Image-Pro Plus version 6.0 and Image J software.

### Immunofluorescence

The tissue sections from patients and mice were deparaffinized and rehydrated, and the antigens were retrieved in boiling 10 mM citrate buffer for 5 min. Then, they were incubated in 10% goat serum to block nonspecific antigens at room temperature. Then, sections were incubated with antibodies against TIM-4 (CST, USA, 1:100), CD68 (Santa Cruz, USA, 1:200), or TGF-β1 (Proteintech, China, 1:500) overnight at 4°C. On day 2, the sections were incubated with anti-mouse IgG-AlexaFluor 594 (Absin, China, 1:500), anti-rabbit IgG-AlexaFluor 488 (Absin, China, 1:500), or anti-rabbit IgG-AlexaFluor 594 (Absin, China, 1:500) secondary antibodies at room temperature for 1 h. Then, they were incubated with DAPI solution for nuclear staining. hNECs and THP-1 cells were fixed and incubated with antibodies against TIM-4 (CST, USA, 1:100), CD68 (Santa Cruz, USA, 1:200), E-cadherin (Proteintech, China, 1:500), and α-SMA (Proteintech, China, 1:500) overnight at 4°C. On day 2, the cells were incubated with fluorescence secondary antibodies and DAPI solution. All results were observed and photographed by fluorescence microscopy and further analyzed by Image-Pro Plus version 6.0 and Image J software.

### Western blotting

Human nasal mucosal tissues, RPMI 2650 cells, and THP-1 cells were homogenized manually and lysed in RIPA lysis buffer containing protease inhibitors and phosphatase inhibitors for 30 min on ice. Protein extracts were loaded into an SDS–PAGE gel and transferred to PVDF membranes (Millipore, USA). Membranes were blocked in 5% fat-free milk for 1.5 h at room temperature, then incubated with primary antibodies against TIM-4 (CST, USA, 1:1000), TGF-β1 (Proteintech, China, 1:1000), E-cadherin (Proteintech, China, 1:5000), α-SMA (Proteintech, China, 1:3000), Vimentin (Proteintech, China, 1:2000), p38 MAPK (CST, USA, 1:1000), p-p38 (CST, USA, 1:1000), or Egr-1 (Proteintech, China, 1:2000) overnight at 4°C. On day 2, they were incubated with anti-rabbit horseradish peroxidase (HRP)-labeled secondary antibody (Proteintech, China, 1:5000) for 1.5 h at room temperature. The results were exposed using a chemiluminescence imaging system and further analyzed by Image J software. The relative expression level of the target protein was evaluated after normalizing to glyceraldehyde-3-phosphate dehydrogenase (GAPDH).

### ELISA

The levels of TGF-β1, MMP2, MMP7, MMP9, and OVA specific IgE (OVA-sIgE) were determined using enzyme-linked immunosorbent assay (ELISA) kits (Bioswamp, Wuhan, China) according to the manufacturer instructions. Briefly, the samples and biotin-conjugated antibodies were applied to ELISA microplates and then detected by using a microplate reader at 450 nm. The concentrations of target protein were calculated by a standard curve according to the manufacturer’s instructions.

### Quantitative RT–PCR

Total RNA was extracted from human nasal mucosal tissues by using TRIzol reagent (Invitrogen, WI, USA). In addition, 1 μg of total RNA was reverse-transcribed to cDNA using a Reverse Transcription Kit (TaKaRa Bio, Beijing, China). PCR reactions were quantified using SYBR Green PCR Master Mix (Invitrogen, WI, USA) in a real-time PCR detection system (Bio-Rad, USA). The primer sequences for *TIM-4* were 5’-ACAGGACAGATGGATGGAATACCC-3’ (forward) and 5’-AGCCTTGTGTGTTTCTGCG-3’ (reverse). Moreover, the primer sequences for *TGF-β1* were 5’-GCCCTGGACACCAACTATTGCT-3’ (forward) and 5’-AGGCTCCAAATGTAGGGGCAGG-3’ (reverse). Relative mRNA levels were analyzed using comparative Ct values (2^−ΔΔCT^), and GAPDH served as a loading control.

### Statistical analysis

All data and graphs were presented as the means ± standard deviation (SD) and were analyzed using SPSS 22.0 software (IBM, Chicago, USA) and GraphPad Prism 8.0 software (San Diego, USA). One-way ANOVA or T test was used to analyze the differences between groups. The correlations between clinical parameters and molecular markers were assessed by Spearman’s rank test. Significance was considered at P<0.05.

## Results

### Increased TIM-4 expression in macrophages in patients with CRSwNP

**Figure 1:**
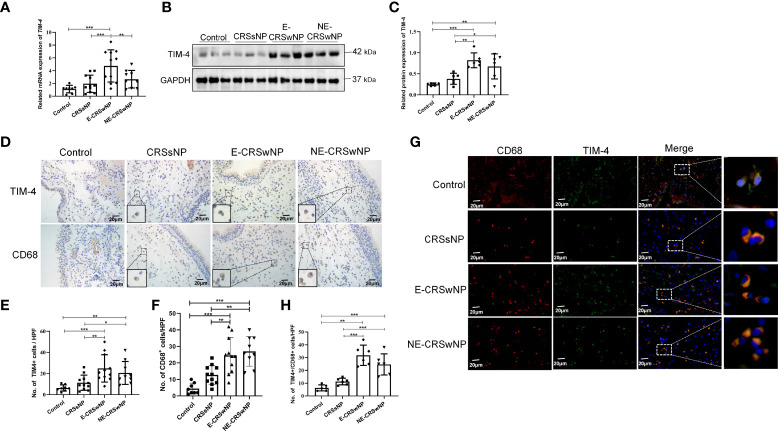
Increased TIM-4 expression in macrophages from patients with CRSwNP. **(A)** The mRNA expression levels of TIM-4 in different human sinonasal mucosae, as detected by quantitative RT–PCR. **(B-E)** Protein expression levels of TIM-4 in the human sinonasal mucosa. **(B)** The protein levels of TIM-4 in the human sinonasal mucosa from the control, CRSsNP, E-CRSwNP, and NE-CRSwNP groups, as detected by Western blot analysis. GAPDH was used as a control. **(C)** Quantitative summary of the relative protein expression of TIM-4. **(D)** The TIM-4^+^ cells and CD68^+^ cells in the human sinonasal mucosa from the control, CRSsNP, E-CRSwNP, and NE-CRSwNP groups were examined by IHC. **(E)** Quantitative summary of the number of TIM-4^+^ cells in the human sinonasal mucosa. **(F)** Quantitative summary of the number of CD68^+^ cells in the human sinonasal mucosa. **(G, H)** Colocalization of TIM-4^+^ and CD68^+^ in the human sinonasal mucosa. **(G)** Double immunofluorescence staining of TIM-4 (green) and CD68^+^ macrophages (red) in the lamina propria of human sinonasal mucosa from the control, CRSsNP, E-CRSwNP, and NE-CRSwNP groups. **(H)** Quantitative summary of the number of TIM-4^+^/CD68^+^ cells in the human sinonasal mucosa. RT–PCR, IHC (control group: n=8; CRSsNP group: n=11; E-CRSwNP group: n=11; NE-CRSwNP group: n=9); Western blot, and immunofluorescence (control group: n=6; CRSsNP group: n=6; E-CRSwNP group: n=6; NE-CRSwNP group: n=6). All of the above representative pictures are shown at a magnification 400×, and the insets show a higher magnification of the selected area. Bars show the mean ± SD. *P<0.05, **P<0.01,***P<0.001.

First, we found that the mRNA expression of TIM-4 was significantly increased in the E-CRSwNP group compared with the control, CRSsNP, and NE-CRSwNP groups (**Figure 1A**). TIM-4 protein level was significantly increased in the CRSwNP group compared with the control and CRSsNP groups **(** **Figures 1B, C**). Consistently, the IHC staining results also demonstrated that TIM-4 expression was increased in both the E-CRSwNP and NE-CRSwNP groups in comparison with the control and CRSsNP groups (**Figures 1D, E**) and was mainly expressed in the lamina propria. Furthermore, we demonstrated that the number of CD68^+^ macrophages was significantly increased in both the E-CRSwNP and NE-CRSwNP groups (**Figures 1D, F**). Immunofluorescence results confirmed the colocalization of TIM-4 and CD68^+^ macrophages. The amount of TIM-4^+^CD68^+^ positive cells were significantly increased in the CRSwNP group (**Figures 1G, H**). In contrast, we also observed the colocalization of TIM-4 and CD11c^+^ dendritic cells (DCs) in the CRSwNP (**Supplementary Figures 1A, B**) and found that the number of TIM4^+^/CD11c^+^ cells was observably less than the TIM-4^+^/CD68^+^ cells in CRSwNP (**Supplementary Figure 1C**). Therefore, it is speculated that the expression of TIM-4 in macrophages may play an important role in CRSwNP.

### TIM-4 positively correlates with EMT in patients with CRS

**Figure 2:**
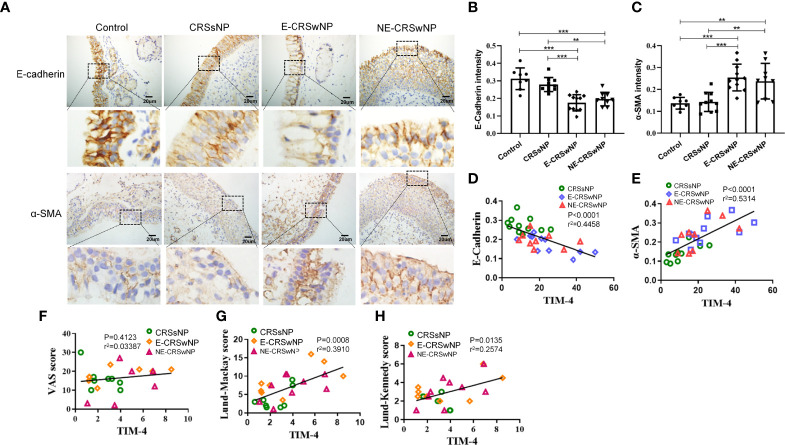
Correlation analysis between TIM-4 and EMT markers in patients with CRS. **(A-C)** The expression of the EMT markers E-cadherin and α-SMA in different human sinonasal mucosal tissues. **(A)** Images show the representative immunostaining of E-cadherin and α-SMA in the sinonasal mucosa of healthy individuals and CRSsNP, E-CRSwNP, and NE-CRSwNP patients by IHC (control group: n=8; CRSsNP group: n=11; E-CRSwNP group: n=11; NE-CRSwNP group: n=9). **(B, C)** Quantitative summary of the intensity of E-cadherin **(B)** and α-SMA **(C)** in the human sinonasal mucosa. **(D, E)** The correlation between TIM-4 and EMT markers E-cadherin and α-SMA. **(F-H)** The correlation between TIM-4 and the clinical parameters of VAS score, Lund–Mackay score and Lund–Kennedy score. The Pearson correlation test was used, and r^2^ represents the coefficient of determination. All of the above representative pictures are shown at a magnification of 400×. Bars show the mean ± SD. **P<0.01,***P<0.001.

EMT markers were analyzed immunohistochemically, and the sinonasal mucosa of the CRSwNP showed typical features of EMT. The intensity of E-cadherin was significantly downregulated in both the E-CRSwNP and NE-CRSwNP groups compared with the control and CRSsNP groups, but there was no difference in the CRSsNP group compared with the control group. In contrast, the expression of the mesenchymal marker α-SMA was elevated in the CRSwNP group compared with the control group (**Figures 2A-C**). Correlation analysis results suggested that TIM-4 expression in CRS was positively correlated with α-SMA and negatively correlated with E-cadherin (**Figures 2D, E**). Moreover, TIM-4 expression in the sinonasal mucosa was correlated with disease severity parameters, which were determined by the Lund–Mackay score or the Lund–Kennedy score, but there was no correlation with VAS score (**Table 1**; **Figures 2F-H**). These results suggest that TIM-4 expression is related to the occurrence and development of disease by promoting EMT.

### TIM-4 mAb alleviates the formation of polypoid lesions in a murine NP model

**Figure 3:**
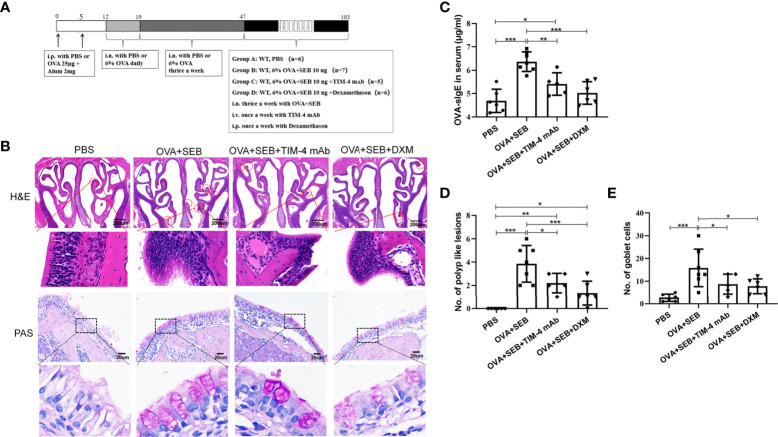
Effects of TIM-4 mAb on polypoid lesion formation in the NP murine model. **(A)** The protocol for the NP murine model. WT mice were sensitized by an i.p. of either PBS or 25 µg of OVA in 2 mg aluminum hydroxide. Then, WT mice were treated with 6% OVA and 10 ng SEB by i.n. administration to induce nasal polyposis, and TIM-4 mAb and dexamethasone were administered to the experimental group. **(B–E)** The effects of TIM-4 mAb on polypoid lesion formation. **(B)** Representative polypoid lesions and typical sinonasal mucosa stained by H&E. Morphology and numbers of goblet cells stained by PAS. The criteria for polypoid lesions were described in the methods section and represented with red circles. **(C)** OVA-specific IgE levels in serum were detected by ELISA. **(D)** Quantitative summary of the number of polypoid lesions. **(E)** Quantitative summary of the number of goblet cells. PBS group: n=6; OVA+SEB group: n=7; OVA+SEB+TIM-4 mAb group: n=5; OVA+SEB+DXM group: n=6; TIM-4 monoclonal antibody: TIM-4 mAb; dexamethasone: DXM; Intraperitoneal: i.p.; intranasal: i.n.; intravenous: i.v. H&E pictures are shown at a magnification of 40×, and PAS, IHC, and immunofluorescence representative pictures are shown at a magnification of 400×. The insets show a higher magnification of the selected area. Bars show the mean ± SD. *P<0.05, **P<0.01, ***P<0.001.

A murine model of NP was established using wild-type mice according to an established reported protocol (**Figure 3A**). OVA+SEB treatment exhibited prominent polypoid lesions and caused an increase in goblet cells and OVA-specific IgE levels compared with the PBS group (**Figures 3B-E**). After NP mice were treated with TIM-4 mAb or dexamethasone, the number of polypoid lesions and goblet cells were significantly reduced compared with the OVA+SEB group (**Figures 3B, D, E**). Additionally, OVA-specific IgE levels in serum were suppressed following TIM-4 mAb or dexamethasone treatment (**Figure 3C**). The results suggest that blocking TIM-4 alleviates the formation of nasal polyps in a murine model of NP.

### TIM-4 interference inhibits the EMT process in the NP model

**Figure 4:**
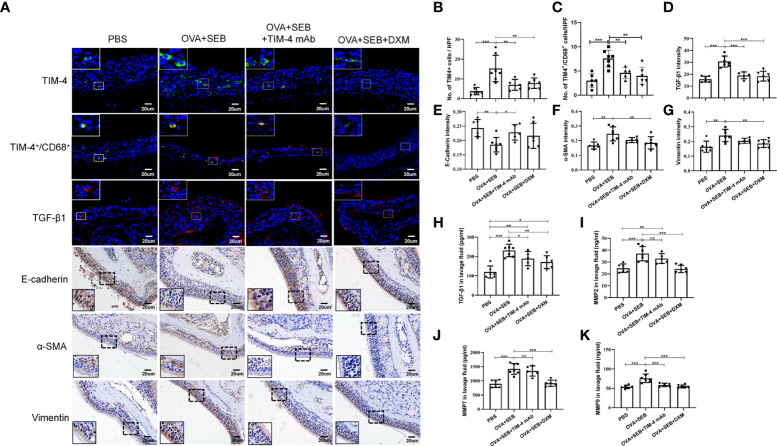
TIM-4 interference inhibits the EMT process in the NP model. **(A)** TIM-4^+^ cells (green), TIM-4^+^/CD68^+^ cells (yellow), and TGF-β1 (red) in the mouse sinonasal mucosa were detected by immunofluorescence. E-cadherin, α-SMA, and Vimentin in the mouse sinonasal mucosa were detected by IHC. **(B)** Quantitative summary of the number of TIM-4^+^ cells in the mouse sinonasal mucosa. **(C)** Quantitative summary of the number of TIM-4^+^/CD68^+^ cells in the mouse sinonasal mucosa. **(D)** Quantitative summary of the intensity of TGF-β1 in the mouse sinonasal mucosa. **(E)** Quantitative summary of the intensity of E-cadherin in the mouse sinonasal mucosa. **(F)** Quantitative summary of the intensity of α-SMA in the mouse sinonasal mucosa. **(G)** Quantitative summary of the intensity of Vimentin in the mouse sinonasal mucosa. **(H-K)** The TGF-β1, MMP2, MMP7, and MMP9 levels in the nasal lavage fluid were detected by ELISA. PBS group: n=6; OVA+SEB group: n=7; OVA+SEB+TIM-4 mAb group: n=5; OVA+SEB+DXM group: n=6. IHC and immunofluorescence representative pictures are shown at a magnification of 400×. The insets show a higher magnification of the selected area. Bars show the mean ± SD. *P<0.05, **P<0.01,***P<0.001. ns, no significance.

In NP model, TIM-4 was increased in the OVA+SEB group and decreased after TIM-4 mAb and dexamethasone treatment (**Figures 4A, B**). The double immunofluorescence result confirmed the colocalization of TIM-4 and CD68^+^ macrophages in the mouse sinonasal mucosa, and the amount of TIM-4^+^/CD68^+^ positive cells were significantly increased in the OVA+SEB group and decreased after TIM-4 mAb and dexamethasone treatment (**Figures 4A, C**. **Supplementary Figure 2A**). To verify the effect of TIM-4 on EMT in the NP model mice, we evaluated the expression of EMT markers by IHC staining. The results confirmed that E-cadherin was significantly decreased in the OVA+SEB group, whereas α-SMA and Vimentin were increased compared with the PBS group. Compared with the OVA+SEB group, E-cadherin was elevated and α-SMA and Vimentin were significantly decreased in the TIM-4 mAb and dexamethasone groups (**Figures 4A, E–G**). Consequently, these findings imply that TIM-4 mAb alleviates the formation of nasal polyps, which may be related to the facilitation of the EMT process.

### TIM-4 mAb influences the TGF-β1 protein level in nasal secretions in the murine model of NP

Previous studies demonstrated that TGF-β1, MMP2, MMP7, and MMP9 could induce EMT in nasal polyps and can be synthesized and secreted by macrophages (11, 31). In the OVA+SEB group, the TGF-β1, MMP2, MMP7, and MMP9 levels were significantly increased in nasal lavage fluid, but only the TGF-β1 levels were decreased after TIM-4 mAb treatment. There was no significant decrease in the MMP2, MMP7, or MMP9 levels (**Figures 4H–K**). Moreover, immunofluorescence result showed that the TGF-β1 intensity was increased in mucosal tissues of the OVA+SEB group and decreased after TIM-4 mAb or dexamethasone treatment (**Figures 4A, D**). These findings suggest an important contribution of TIM-4 to activate TGF-β1 signaling, which might mediate the occurrence of EMT.

### TIM-4 in macrophages contributes to EMT in hNECs

**Figure 5:**
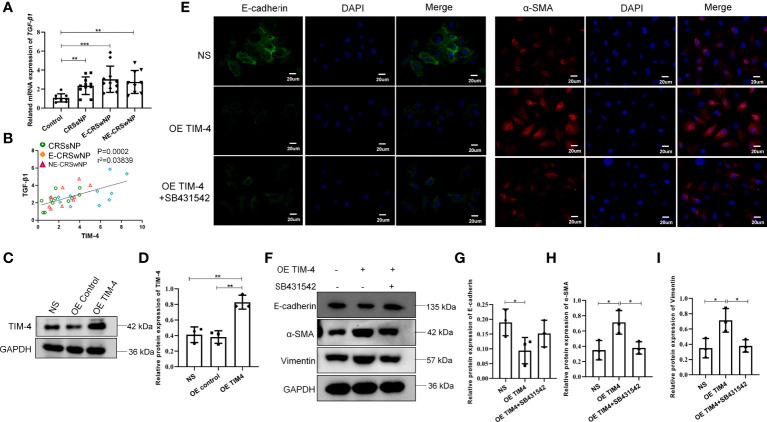
TIM-4 expression in macrophages contributes to EMT of nasal epithelial cells. **(A)** The mRNA expression levels of TGF-β1 in different human sinonasal mucosa, as detected by quantitative RT–PCR. **(B)** The correlation between TIM-4 and TGF-β1. **(C)** TIM-4 overexpression in THP-1 cells was measured by Western blot analysis. GAPDH was used as a control. **(D)** Quantitative summary of the relative protein expression of TIM-4. **(E)** hNECs were cultured with OE TIM-4 THP-1 cells transfected with lentivirus and treated with 10 μmol/L TGF-β1 inhibitor (SB431542) for 24 h. The expression of EMT markers E-cadherin and α-SMA was detected by immunofluorescence. **(F)** The OE TIM-4 THP-1 cells and RPMI2650 were cocultured and treated with 10 μmol/L TGF-β1 inhibitor (SB431542) for 24 h, and the expression of EMT markers E-cadherin, α-SMA, and Vimentin were detected by Western blot analysis. The intensity values of the bands were normalized to GAPDH expression. **(G)** Quantitative summary of the relative protein expression of E-cadherin. **(H)** Quantitative summary of the relative protein expression of α-SMA. **(I)** Quantitative summary of the relative protein expression of Vimentin. Bars show the mean ± SD. *P<0.05, **P<0.01, ***P<0.001. Representative immunofluorescence images are shown at 400× magnification.

Then, we further analyzed the relationship between TIM-4 and TGF-β1 in clinical samples and found that the mRNA expression of TGF-β1 was significantly increased in both the CRSwNP and CRSsNP groups compared with the control group (**Figure 5A**). The correlation analysis suggested that the TIM-4 expression in the sinonasal mucosa was positively correlated with TGF-β1 (**Figure 5B**). To determine the relationship between TIM-4 and TGF-β1, THP-1 cells were treated with OE TIM-4, and then cocultured with hNECs. We measured the transcriptional activity of TIM-4 with lentivirus vector to overexpress TIM-4 in THP-1 cells (**Figures 5C, D**). The results showed that E-cadherin was decreased in hNECs, and α-SMA and Vimentin were increased in hNECs compared with the control group (**Figure 5E**). Then, after the TGF-β1 inhibitor SB431542 was used in the coculture systems of THP-1 cells and RPMI 2650 cells, the EMT markers were partly blocked (**Figures 5F-I**). These results indicate that TIM-4 in macrophages mediated EMT in hNECs.

### Tissue extracts of CRSwNP promote the expression of TIM-4 and TGF-β1 in macrophages

**Figure 6:**
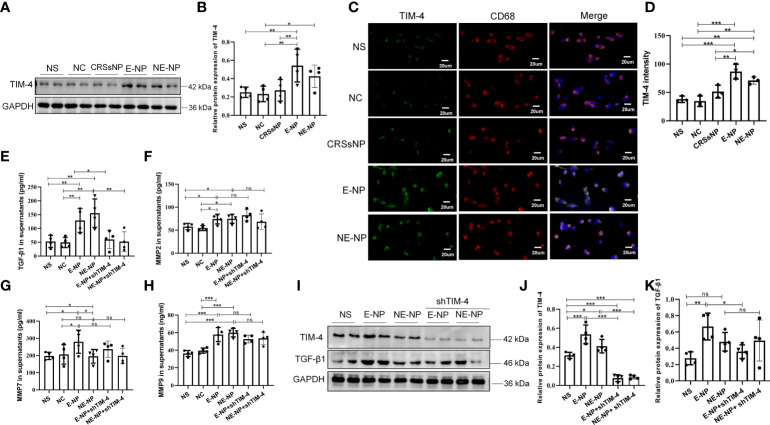
Tissue extracts promote the expression of TIM-4, TGF-β1, MMP2, MMP7, and MMP9 in macrophages. **(A)** TIM-4 protein expression in THP-1 cells treated with nasal tissue extracts from healthy subjects (NC group) and patients with CRSsNP, E-CRSwNP (E-NP group), and NE-CRSwNP (E-NP group) for 24 h was measured by Western blot analysis. For each group, n=4. **(B)** Quantitative summary of the relative protein expression of TIM-4. **(C)** TIM-4 expression in THP-1 cells treated with nasal tissue extracts from NC, CRSsNP, E-NP, and NE-CRSwNP groups for 24 h was measured by immunofluorescence. Images show representative immunostaining for TIM-4 (green) and CD68^+^ macrophages (red). For each group, n=3. **(D)** Quantitative summary of the intensity of TIM-4 in THP-1 cells. **(E-H)** The TGF-β1, MMP2, MMP7, and MMP9 levels in THP-1 cell supernatants after treatment with nasal tissue extracts from healthy people NC, CRSsNP, E-NP, and NE-CRSwNP groups for 24 h were detected by ELISA. For each group, n=4. **(I)** The protein levels of TIM-4 and TGF-β1 in THP-1 cells were measured by Western blot analysis after treatment with nasal tissue extracts and TIM-4 interference, and the intensity values of the bands were normalized to GAPDH expression. For each group: n=4. **(J)** Quantitative summary of the relative protein expression of TIM-4. **(K)** Quantitative summary of the relative protein expression of TGF-β1. Representative immunofluorescence images are shown at 400× magnification. Bars show the mean ± SD. *P<0.05, **P<0.01,***P<0.001. ns, no significance.

THP-1 cells were treated with different tissue extracts from patients with CRSwNP and CRSsNP and healthy subjects, and then the expression of TIM-4 was measured. The results showed that TIM-4 was upregulated after treatment with the CRSwNP extracts, but there was no change with the CRSsNP or control extracts (**Figures 6A-D**). The results suggest that the inflammatory microenvironment of CRSwNP promotes the expression of TIM-4. Then, we examined expression of the TGF-β1, MMP2, MMP7, and MMP9. These cytokines can be synthesized and secreted by macrophages and are involved in EMT. We found that CRSwNP tissue extracts increased the levels of TGF-β1, MMP2, MMP7, and MMP9 in the supernatants of THP-1 cells (**Figures 6E-H**). We further focused on the associations between TIM-4 and these factors and found that only TGF-β1 was suppressed by TIM-4 interference, and no change in the MMPs was discovered (**Figures 6E-K**).

### The ROS/p38 MAPK/Egr-1 pathway participates in the process of TIM-4–mediated TGF-β1 synthesis and secretion in macrophages

**Figure 7:**
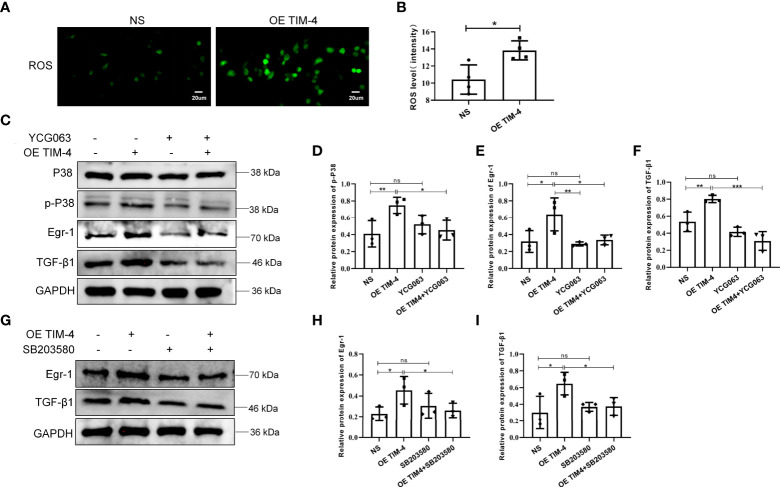
The ROS/p38 MAPK/Egr-1 pathway participates in the process of TIM-4-mediated TGF-β1 upregulation in macrophages. **(A, B)** ROS levels in OE TIM-4 THP-1 cells and control cells were measured by the fluorescence probe DCFH-DA; **(C-F)** Normal or OE TIM-4 THP-1 cells were treated with or without a ROS inhibitor (YCG063), and then the expression of Egr-1, phospho-p38, and TGF-β1 were analyzed by Western blot analysis; **(G-I)** To confirm the role of p38 MAPK in the ROS-dependent signaling pathway, normal or OE TIM-4 THP-1 cells were treated with a p38 inhibitor (SB203580). Then the expression of Egr-1 and TGF-β1 were analyzed by Western blot analysis. The intensity values of the bands were normalized to GAPDH expression. For each group, n=3; Representative immunofluorescence images are shown at 400× magnification. Bars show the mean ± SD. *P<0.05, **P<0.01,***P<0.001. ns, no significance.

A previous study found that the production of TGF-β1 may depend on the accumulation of ROS (25). In our study, we showed that THP-1 cells, treated with the OE TIM-4, produced significantly more ROS than control cells (**Figures 7A, B**). To explore whether ROS participates in the regulation of TGF-β1 by TIM-4, the ROS-mediated pathway was explored. We found that TIM-4 overexpression increased the phosphorylation of p38 MAPK and the expression of Egr-1 and TGF-β1, and a ROS inhibitor (YCG063) reversed these effects (**Figures 7C–F**). Furthermore, a p38/MAPK inhibitor (SB203580) notably decreased the expression of Egr-1 and TGF-β1 that were induced by the OE TIM-4 (**Figures 7G–I**). Therefore, the above results show that TIM-4 upregulates TGF-β1 expression *via* the ROS/p38 MAPK/Egr-1 pathway.

## Discussion

CRSwNP is considered a chronic inflammation of the nasal mucosa and is associated with a variety of cells and inflammatory mediators. However, the exact pathogenesis of it remains unclear. EMT is not only related to asthma (32) but also plays a key role in the pathogenesis of CRSwNP (33). With the aggravation of EMT, the formation of the subepithelial fibrous tissue and thickening of basement membrane are more obvious in the sinus epithelial area. Several studies have also demonstrated that the inhibition of EMT progression sufficiently blocks NP formation (3, 4, 6, 30). In our study, the expression of E-cadherin was significantly decreased, and the expression of α-SMA and Vimentin were significantly increased in the CRSwNP patients and the NP murine model. Furthermore, EMT was positively correlated with the severity of the disease, which is consistent with previous research (4, 34–36). Additionally, we observed that the increased expression of TIM-4 in macrophages promotes EMT in NP formation *via* various kinds of experiments for the first time. TIM-4 was negatively related to E-cadherin but positively correlated with α-SMA. Meanwhile, increased expression of TIM-4 in macrophages promotes EMT in hNECs *in vitro*. Despite the lack of relevant studies, a few reports in oncology support our observation. Recently, Chen et al. demonstrated that the N-glycosylation of TIM-4 promotes EMT in non-small-cell lung cancer (37). Some studies also reported that the effects of TIM-4 have been implicated in respiratory airway diseases such as allergy and asthma (38). Therefore, identifying the role of TIM-4 in macrophages is critical for further elucidating the mechanism of CRSwNP.

It is well known that macrophages play a crucial role in tissue homeostasis and inflammation. After inflammatory injury, macrophages are recruited to release a powerful mediators that trigger EMT. In addition, macrophages are also involved in tissue remodeling in the pathogenesis of CRSwNP and significantly correlated with the disease severity of CRSwNP (11, 15). But the impact factors that involved in macrophage-mediated tissue remodeling in CRSwNP remain to be determined. TIM-4 has been recognized as an important immune regulator and was involved in many immune-related diseases, including allergy, asthma, autoimmune disease, and cancer (38, 39). Here, we found that the amount of CD68^+^ macrophages and the expression of TIM-4 in CD68^+^ macrophages were increased in both the E-CRSwNP and NE-CRSwNP groups. Because TIM-4 is selectively expressed in APCs, especially in macrophages, the increased number of macrophages may directly result in the increased expression of TIM-4. However, whether the increased production of TIM-4 results an increased number of macrophage, it needs to be explored in the future. Moreover, our findings might indicate that TIM-4^+^ macrophages are strongly correlated with the phenotype, irrespective of the inflammatory endotype. Besides macrophages, studies also found that TIM-4 may also be expressed in DCs. We also made an evaluation on the relationship between TIM-4 and DCs in human nasal tissues and found that the colocalization of TIM-4 and CD11c^+^ DCs in the CRSwNP, and TIM4^+^/CD11c^+^ cells are less than the TIM-4^+^/CD68^+^ cells in CRSwNP. We suspected that the differential expression of TIM-4 in macrophages and DCs may be closely related to the microenvironment of CRSwNP.

At the same time, we also found that the expression of TIM-4 in CRSwNP was higher than that in CRSsNP, which suggested that different microenvironments in CRSwNP or CRSsNP patients influence TIM-4 levels. Previous studies confirmed that the specific microenvironment regulates TIM-4 expression. For instance, TIM-4 expression was downregulated in food allergies (40), but its expression was increased in cancer (41), nonalcohol fatty liver disease (42), allergic rhinitis (43), and asthma (19). Additionally, the expression of TIM-4 in macrophages can be stimulated by various factors in the tumor or nonalcohol fatty liver microenvironment (42, 44), such as LPS, danger-associated molecular patterns, ConA, and cytokines. Similarly, there are a large number of inflammatory factors in CRSwNP, and we found that the expression of TIM-4 was increased in THP-1 cells after treatment with tissue extracts from patients with CRSwNP. However, the specific factors enhancing TIM-4 expression in macrophages under CRSwNP have not yet been determined, and a further investigation is needed to elucidate these mechanisms. We speculate that the elevated expression of TIM-4 in the macrophages of CRSwNP may be caused by the costimulation of multiple factors.

Previous studies have demonstrated that macrophages are the predominant cellular sources of MMPs and TGF-β1, which are considered to be causative cytokines for EMT in CRSwNP (10, 13, 45, 46). The functions of macrophages are achieved by responding to different conditions (16, 47, 48). In pulmonary fibrosis, TGF-β1 secreted by macrophages can be stimulated by Th2 cytokines and other cytokines and drive the airway remodeling process (49). In a model of vascular injury, MMPs produced by macrophages also can be mediated by several factors, such as TNF-α and adenosine (50). In addition, TIM-4 has been found to affect the release of cytokines, such as TNF-α, IL-10, TGF-β1, and PGE2 from macrophages (25, 51). So we speculated that TIM-4 might regulate the secretory function of macrophage in CRSwNP. Here, we found that the TGF-β1, MMP2, MMP7, and MMP9 levels were increased in the supernatants from THP-1 cells treated with tissue extracts from patients with CRSwNP, but only TGF-β1 was suppressed by TIM-4 inhibition. Simultaneously, we further confirmed that TIM-4 mediated the EMT through TGF-β1 in the coculture procedures of macrophages and hNECs. The results suggest that TGF-β1 synthesis and secretion in macrophages partly depend on TIM-4, and MMPs may be mediated by other factors. After being secreted out of the macrophages, the TGF-β1 could bind to TGF-β1 receptors on epithelial cells and promote EMT through TGF-β1/Smad pathway (52).

Previous studies have confirmed that hypoxia induces EMT in CRSwNP (3, 9). It also induces hypoxia inducible factor-α expression, which triggers the secretion of tissue remodeling markers, such as TGF-β1 and MMPs. Additionally, a study on macrophages suggested that the regulatory effects of TIM-4 on TGF-β1 synthesis and secretion are related to mitophagy (25), and mitochondrial dysfunction is accompanied by the production of a large number of reactive oxygen species (ROS). ROS is also considered a secondary messenger that affects macrophages activation. And it is involved in a various chronic inflammatory diseases by many endogenous and exogenous insults (53). It has been reported that TIM-4 in macrophages manifests high-mitochondria activity, presents higher levels of oxidative phosphorylation, and induces accumulation of mitochondria related ROS, which may be related to the increased density of LC-3II in autophagosomes *via* mTORC1/ULK1 pathway (54). Another study confirmed that TIM-4 in macrophages mediates the production of mitochondrial ROS *via* Akt1 pathway, and resulting in mitophagy during liver fibrosis (25). The structure and function of mitochondria of macrophages are markedly altered in the context of chronic inflammation, which may cause oxidative phosphorylation metabolic dysfunction, thereby regulating the function of macrophages. Therefore, we speculate that TIM-4 might be involved in regulating the process of CRSwNP formation by affecting the production of ROS. Indeed, in our experiments, TIM-4 overexpression caused an increase in ROS in THP-1 cells. Endogenous ROS can indirectly damage DNA, lipids, and proteins and activates the transcriptional factors through various pathways during inflammation. Egr-1, as a multifunctional transcription factor, is susceptible to hypoxia and upregulates the expression of transcription factor during EMT (55) and can be activated by ROS (56, 57). In addition, Egr-1 can transactivate TGF-β1 and is regulated by various cellular kinases, such as p38/MAPK (55). In this study, we found that ROS, p38/MAPK, Egr-1, and TGF-β1 levels were increased in THP-1 cells after overexpressing TIM-4. By blocking ROS or p38/MAPK, the expression of Egr-1 and TGF-β1 were inhibited, which is consistent with Li and Son’s reports (58, 59). The results indicate that the effect of TIM-4 on synthesis and secretion of TGF-β1 by macrophages might be predominantly mediated by ROS/p38 MAPK/Egr-1. A large amount of evidence demonstrated that ROS is closely related to macrophages glucose metabolism and that scavenging of ROS could improve macrophage-induced inflammation (35). Perhaps, the regulation of macrophages is closely related to the metabolism in CRSwNP. However, the specific mechanism in this process needs further study.

Although we have preliminarily confirmed that TIM-4 in macrophages contributes to nasal polyp formation through TGF-β1–mediated EMT *in vivo* and *in vitro*, there are some limitations in the present study. First, in our study, we confirmed that TIM-4 was highly selectively expressed in macrophages, which is consistent with previous reports, but the role of TIM-4 on other immune cells has not been clearly defined in CRSwNP and a murine model, it could not be completely ruled out that TIM-4 is involved in the formation of nasal polyps by regulating other immune cells. Therefore, further experiments with macrophage depletion *in vivo* are needed to verify the role of TIM-4 in nasal polyps formation in the future. Second, we preliminarily confirmed the regulation role of TIM-4 on the secretion of TGF-β1 by macrophages, but studies have shown that macrophages are not the entire source of TGF-β1 *in vivo*, and it may be synthesized and secreted by other cells. The role of TIM-4 in regulating TGF-β1 through other cells or other pathways also needs future studies. Third, TIM-1 and phosphatidylserine are the endogenous ligands of TIM-4, they could bind to TIM-4 and regulate the function of macrophages. Phosphatidylserine/TIM-4 interaction enhances phagocytosing activity of macrophages independent of the classical engulfment pathway, and probably relies on other cell-surface receptors for signaling to maintain the homeostasis (60). In addition, TIM-1/TIM-4 interaction regulates T cell proliferation and modulates Th1/Th2 balance in asthma, allergy, and autoimmunity (61, 62). Another study demonstrated that cross-linking TIM-1 and TIM-4 in macrophages results in TNF-α/IL-6 secretion and contributes to ischemia reperfusion-induced liver damage (63). However, there is a lack of relevant studies confirming that the interaction of TIM-1/TIM-4 affects the ROS-dependent P38/MAPK pathway. Whether TIM-4 ligands are highly expressed in CRSwNP and whether they may also be involved in the occurrence of nasal polyps by stimulating TIM-4 in macrophages need further research.

**Figure 8:**
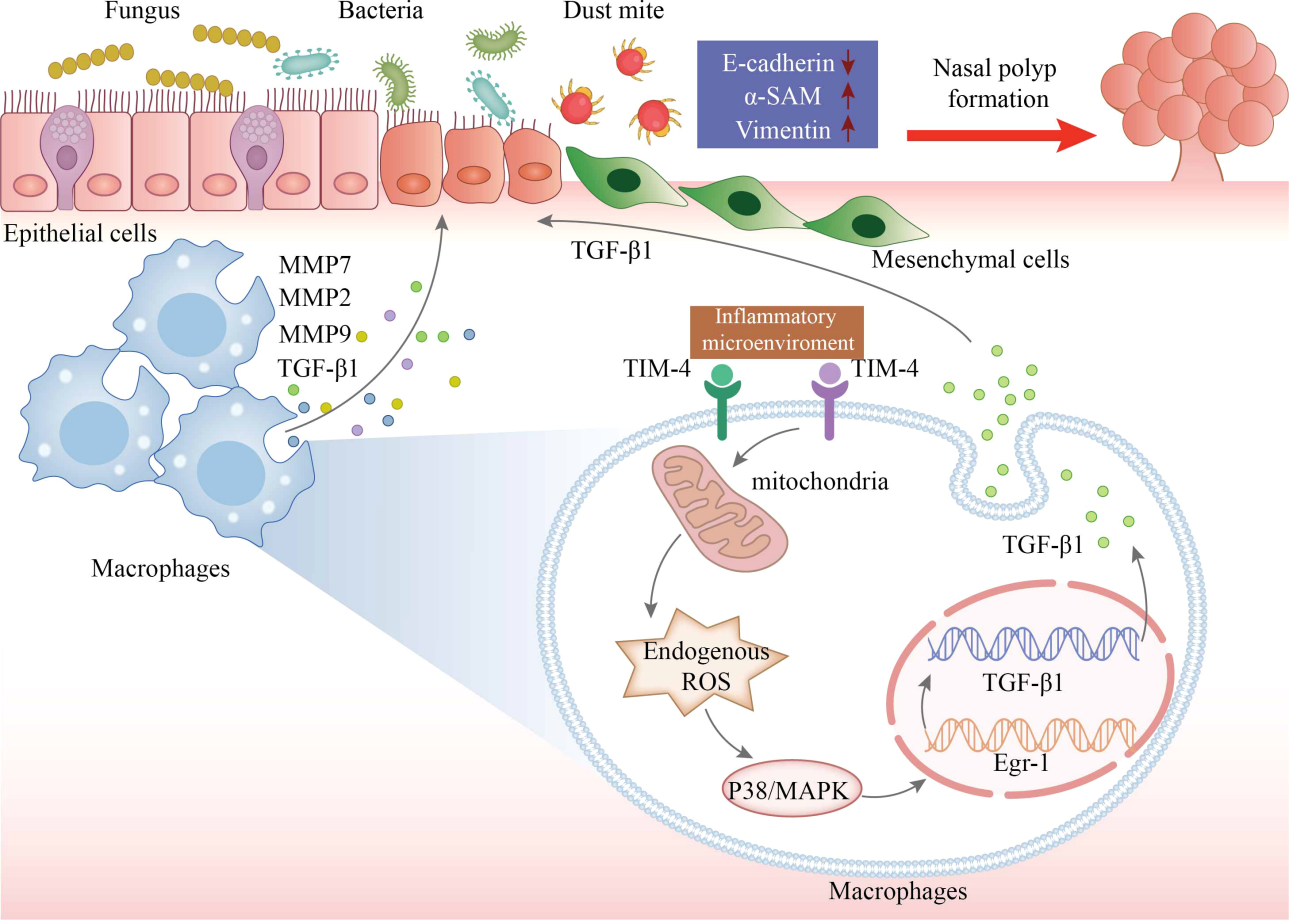
The schematic model for TIM-4 in macrophages mediates TGF-β1 secretion *via* the ROS/p38 MAPK/Egr-1 pathway to regulate nasal polyp formation. The increased production of TIM-4 in macrophages is stimulated by inflammatory microenvironment of CRSwNP. TIM-4 boosts the activity of the secondary messenger ROS. The activation of ROS promotes P38 MAPK phosphorylation and then increases the transcriptional activity of Egr-1. The cascade increases the transcription and translation of TGF-β1 in macrophages, resulting in an increase of TGF-β1 secretion, which culminates in promoted EMT in nasal epithelial cells. In conclusion, both increased accumulation of macrophages and increased TIM-4 production by macrophages may result in ROS-dependent activation of p38/MAPK/Egr-1 cascade, which contributes to nasal polyp formation.

Taken together, we evaluated a previously unrecognized link between TIM-4 and CRSwNP for the first time. The increased production of TIM-4 in macrophages may contribute to regulating the EMT process in CRSwNP by facilitating the production of TGF-β1 *via* ROS/p38 MAPK/Egr-1 pathway (**Figure 8**). Therefore, TIM-4 may represent a potential therapeutic target for nasal polyposis.

## Data availability statement

The original contributions presented in the study are included in the article/**Supplementary Material**. Further inquiries can be directed to the corresponding author.

## Ethics statement

The studies involving human participants were reviewed and approved by Ethics Committee of Renmin Hospital of Wuhan University. The patients/participants provided their written informed consent to participate in this study. The animal study was reviewed and approved by Animal Ethics Committee of Renmin Hospital of Wuhan University.

## Author contributions

YX and ZT provided funding and supervision and reviewed and edited the manuscript. DQ and PL designed and performed experiments, analyzed the data and contributed to writing the original draft of the manuscript. KL, HZ, JJ, WG, SC, and JH assessed and recorded the patients’ health information and collected the samples from the participants. WF provided analytical tools and reagents. All authors discussed the results and commented on the manuscript. All authors contributed to the article and approved the submitted version.

## Funding

This work was supported by grants from the National Natural Science Foundation of China (NSFC): No. 81770986 (YX); No. 82071017 (YX) and the Fundamental Research Funds for the Central Universities: No. 2042020kf1044 (YX).

## Conflict of interest

The authors declare that the research was conducted in the absence of any commercial or financial relationships that could be construed as a potential conflict of interest.

## Publisher’s note

All claims expressed in this article are solely those of the authors and do not necessarily represent those of their affiliated organizations, or those of the publisher, the editors and the reviewers. Any product that may be evaluated in this article, or claim that may be made by its manufacturer, is not guaranteed or endorsed by the publisher.

## Abbreviations

CRS, chronic rhinosinusitis; CRSwNP, chronic rhinosinusitis with nasal polyps; E-CRSwNP, eosinophilic CRSwNP; NE-CRSwNP, noneosinophilic CRSwNP; TIM-4, T-cell immunoglobulin domain and mucin domain; EMT, epithelial to mesenchymal transition; TGF-β1, transforming growth factor-beta1; hNECs, human nasal epithelial cells; MMPs, matrix metalloproteinases; APC, antigen-presenting cells; ESS, endoscopic sinus surgery; DXM, dexamethasone; i.n., intranasal; i.p., intraperitoneal; i.v., intravenous; NP, nasal polyp; GAPDH, glyceraldehyde-3-phosphate dehydrogenase; ns, no significance.
