# Supplementary material for: TIM-4 in macrophages contributes to nasal polyp formation through the TGF-β1–mediated epithelial to mesenchymal transition in nasal epithelial cells
Source: Front Immunol. 2022 Aug 5;13:941608. doi: 10.3389/fimmu.2022.941608 (PMC9389014; doi:10.3389/fimmu.2022.941608)
Supplement: Supplementary file 1 [file DataSheet_1.docx]

**Supplementary Figure 1**


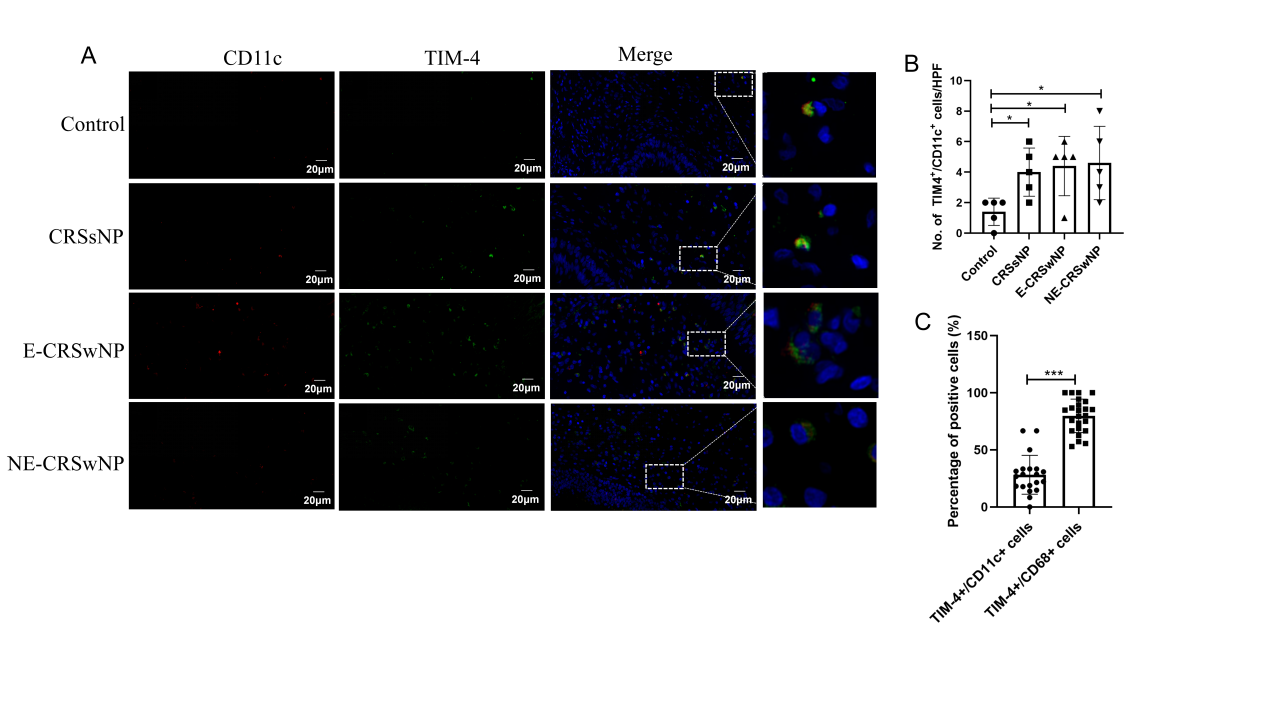


**Supplementary Figure 1** The expression of TIM-4 in DCs from patients with CRSwNP

(A) Double immunofluorescence staining of TIM-4 (green) and CD11c^+^ DCs (red) in the lamina propria of human sinonasal mucosa from the control, CRSsNP, E-CRSwNP, NE-CRSwNP groups. (B) Quantitative summary of the number of TIM-4^+^/CD11c^+^ cells in the human sinonasal mucosa. (3) Mean percentages of TIM-4^+^/CD68^+^ macrophages and TIM-4^+^/CD11c^+^ DCs cells accounting for the TIM-4^+^ cells. All of the above representative pictures are shown at a magnification 400×, and the insets show a higher magnification of the selected area. Bars show the mean±SD. *P<0.05, ***P<0.001.
