# Supplementary material for: TIM-4 in macrophages contributes to nasal polyp formation through the TGF-β1–mediated epithelial to mesenchymal transition in nasal epithelial cells
Source: Front Immunol. 2022 Aug 5;13:941608. doi: 10.3389/fimmu.2022.941608 (PMC9389014; doi:10.3389/fimmu.2022.941608)
Supplement: Supplementary file 2 [file DataSheet_2.docx]

**Supplementary Figure 2**

**
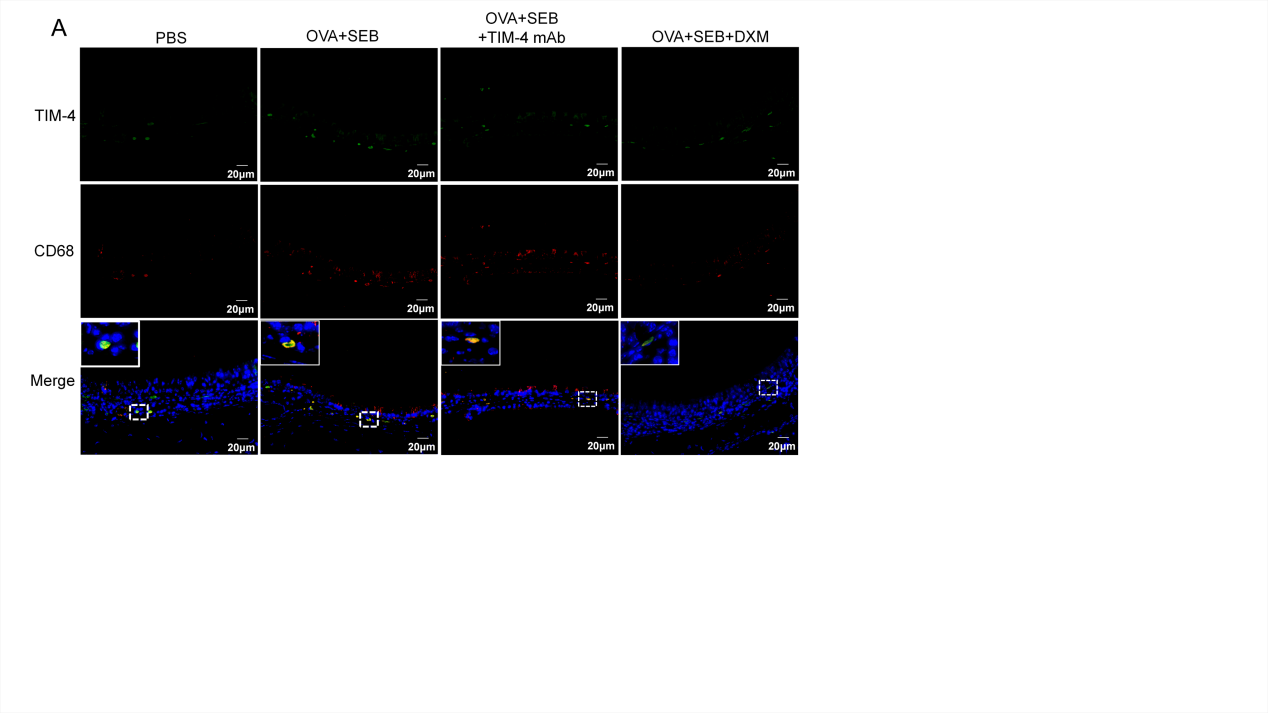
**

**Supplementary Figure 2 Colocalization of TIM-4^+^ and CD68^+^ in the NP murine model**

1. Double immunofluorescence staining of TIM-4 (green) and CD68 (red) in the mouse sinonasal mucosa. All of the above representative pictures are shown at a magnification of 400×; The insets show a higher magnification of the selected area.
